# A commentary on “Brain–computer interfaces: the innovative to unlocking neurological conditions”

**DOI:** 10.1097/JS9.0000000000003094

**Published:** 2025-07-18

**Authors:** Chen Fan, Yuan Ding, Hongri Zhang

**Affiliations:** a Department of Neurosurgery, The First Affiliated Hospital, and College of Clinical Medical of Henan University of Science and Technology, Luoyang, Henan, China; b Department of Traditional Chinese Medicine Rehabilitation, The People’s Hospital of Atushi City, Xinjiang, China; c Department of Neurological Rehabilitation, Kunshan Rehabilitation Hospital, Kunshan, Jiangsu, China

*Dear Editor,*

We read with great interest the recent article titled “Brain–computer interfaces: the innovative key to unlocking neurological conditions”^[1]^ published in the International Journal of Surgery. An extensive discussion of the history of development of BCI technology opens the document, which then delves into its uses in treating neurological disorders before looking at potential future research avenues. Future studies must, however, address a number of important concerns.

First, a number of case studies and randomized controlled trials (RCTs) are cited in the publication; however, these studies typically lack long-term follow-up data and have small sample sizes. For instance, when discussing the rehabilitation outcomes of BCI for patients with spinal cord injuries (SCI), only a few studies are cited, and the varying responses to different levels of injury are not addressed^[2]^. Moreover, the paper fails to adequately analyze the failures or limitations of BCI technology, which may lead readers to have overly optimistic expectations about its practical applications. The lack of clinical data undermines the empirical foundation of the paper, limiting the generalizability of its conclusions.

Second, the report does not provide thorough explanations of the particulars of BCI technology implementation. For instance, when discussing signal processing and classification algorithms, it only briefly mentions methods such as Support Vector Machines (SVM) and Linear Discriminant Analysis (LDA), without delving into parameter optimization or performance comparisons in practical applications^[3]^. Furthermore, the analysis of the advantages and disadvantages of invasive versus non-invasive BCI is rather general, without specifying the applicability of different technologies in various clinical settings. The lack of technical details may make it difficult for engineering professionals to derive practical reference information from the paper.

Third, the document does not provide precise technological roadmaps or staged targets; instead, it provides a broad overview of the future evolution of BCI technology. For instance, while it mentions “bidirectional brain-computer interfaces” and “high-performance BCI” as future directions, it does not specify the key technological challenges that need to be overcome to achieve these goals. Furthermore, the paper does not explore the potential for integrating BCI technology with other emerging technologies, such as quantum computing or brain-cloud interfaces. The ambiguity about future directions may reduce the paper’s practical value for researchers.

Overall, the paper provides a comprehensive overview of the clinical application of BCI technology. However, it has notable shortcomings in the depth of literature, clinical data, technical details, ethical considerations, future directions, and the presentation of charts. Future research can build on this foundation by further refining the technical implementation, adding more clinical evidence, and enhancing discussions on ethical and safety issues. The TITAN guideline, which demands openness in the reporting of artificial intelligence, was mentioned in connection with all of this^[4]^.

## Data Availability

No data was used in this Letter to the Editor.

## References

[1] Zhang H Jiao L Yang S. Brain-computer interfaces: the innovative key to unlocking neurological conditions. Int J Surg 2024;110:5745–62. 39166947 10.1097/JS9.0000000000002022 PMC11392146

[2] Feigin VL Abajobir AA Abate KH Global, regional, and national burden of neurological disorders during 1990–2015: a systematic analysis for the Global Burden of Disease Study 2015. Lancet Neurol 2017;16:877–97. 28931491 10.1016/S1474-4422(17)30299-5 PMC5641502

[3] Flesher SN Downey JE Weiss JM. A brain-computer interface that evokes tactile sensations improves robotic arm control. Science 2021;372:831–36. 34016775 10.1126/science.abd0380 PMC8715714

[4] Agha RA Mathew G Rashid R. Transparency In The reporting of Artificial INtelligence – the TITAN guideline. Prem J Sci 2025;10:100082.

